# A Review of Current Evidence on the Relationship between Phosphate Metabolism and Metabolic Syndrome

**DOI:** 10.3390/nu14214525

**Published:** 2022-10-27

**Authors:** Sok Kuan Wong

**Affiliations:** Department of Pharmacology, Faculty of Medicine, Universiti Kebangsaan Malaysia, Jalan Yaacob Latif, Bandar Tun Razak, Cheras, Kuala Lumpur 56000, Malaysia; jocylnwsk@gmail.com; Tel.: +60-3-9145-9566

**Keywords:** diabetes, dyslipidaemia, parathyroid hormone, FGF-23, obesity

## Abstract

Phosphorus, present as phosphate in biological systems, is an essential mineral for various biological activities and biochemical processes. Numerous studies have indicated that disturbed phosphate balance may contribute to the development of metabolic syndrome (MetS). However, no consistent result was found on the association between phosphorus intake and serum phosphate concentration with MetS. It is believed that both positive and negative impacts of phosphorus/phosphate co-exist in parallel during MetS condition. Reduced phosphate level contributed to the development of obesity and hyperglycaemia. Low phosphate is believed to compromise energy production, reduce exercise capacity, increase food ingestion, and impair glucose metabolism. On the other hand, the effects of phosphorus/phosphate on hypertension are rather complex depending on the source of phosphorus and subjects’ health conditions. Phosphorus excess activates sympathetic nervous system, renin-angiotensin-aldosterone system, and induces hormonal changes under pathological conditions, contributing to the blood pressure-rising effects. For lipid metabolism, adequate phosphate content ensures a balanced lipid profile through regulation of fatty acid biosynthesis, oxidation, and bile acid excretion. In conclusion, phosphate metabolism serves as a potential key feature for the development and progression of MetS. Dietary phosphorus and serum phosphate level should be under close monitoring for the management of MetS.

## 1. Introduction

Phosphorus is the second most abundant mineral with widespread extracellular and intracellular distribution in humans, constituting about 1% of total body weight [1]. It mainly exists in the organic form as a complex with carbohydrates, lipids, and proteins with small amount in the form of unbound inorganic phosphate in the extracellular fluid space. The phosphate concentration in serum is tightly maintained between 2.5 to 4.5 mg/dL (0.80 to 1.45 mmol/L) in healthy adults [2] and slightly higher in children depending on their age [3]. Phosphorus has diverse functions in (a) bones and teeth formation, (b) deoxyribonucleic acid and ribonucleic acid synthesis, (c) energy production and storage, (d) protein synthesis for cells and tissues growth, maintenance, and repair, (e) maintaining blood pH, as well as (f) intercellular signalling for gene expression via phosphorylation/dephosphorylation [1,4]. The recommended dietary allowance for phosphorus is 700 mg/day for adults whereas 500–1250 mg/day for children and adolescents aged 6–19 years old [5].

The multifaceted roles of phosphorus suggest its significance in maintaining the physiological processes in humans. The disturbance in phosphate metabolism, either a deficiency or excess of phosphate, may represent a key feature of metabolic syndrome (MetS). By definition, MetS is a combination of at least three clinical manifestations including central obesity, hyperglycaemia, hypertension, hypertriglyceridemia, and low high-density lipoprotein cholesterol (HDL-C) [6]. Both experimental and clinical studies have revealed that alteration of phosphate level affects glucose and lipid metabolism [7]. Phosphate promotes the phosphorylation of carbohydrate intermediate in glycolysis and glycogenesis [8,9]. Genes involved in fatty acid oxidation are upregulated whereas genes involved in fatty acid and cholesterol biosynthesis are downregulated in response to high phosphate diet [10]. Researches investigating the net outcome of phosphate on MetS yielded heterogenous results with positive [11,12], negative [13,14] or negligible association [15]. Thus, the effects of phosphate in each MetS component need to be scrutinised for better understanding on this topic.

In present review, the available evidence on the relationship between phosphorus intake and serum phosphate level with MetS and its individual components are collated. The underlying mechanisms suggesting the possible link between phosphate metabolism with development of obesity, hyperglycaemia, hypertension, and dyslipidaemia that characterise MetS are also discussed. This review provides an overview of current understanding on the effects of phosphate in the pathophysiology of MetS.

## 2. Search Strategy

Literature search was performed using PubMed and Scopus databases using appropriate keywords, including “phosphate”, “phosphorus”, “metabolic syndrome”, “obesity”, “diabetes”, “hypertension”, and “dyslipidaemia”. The studies published from inception to 31 August 2022 were identified. The inclusion criteria adopted for the literature analysis are original research studies reporting the relationship between phosphorus intake/supplementation and circulating phosphate level on MetS and its individual components in animals and humans. Reviews, editorials, commentary, articles not published in English language, and irrelevant articles are excluded. A total of 44 articles meeting the criteria were included in this review.

## 3. The Association between Phosphate Metabolism with MetS and Its Associated Conditions

### 3.1. Phosphate Metabolism and MetS

MetS appears as complex abnormalities of increased abdominal circumference, fasting blood glucose (FBG), blood pressure, and disordered lipid metabolism. In a standardised preclinical setting, it was found that male rats fed with high-carbohydrate high-fat diet for 20 weeks displayed MetS [16,17,18] with elevated parathyroid hormone (PTH) and fibroblast growth factor-23 (FGF-23), but lowered phosphate-regulating gene with homologies to endopeptidases on the X chromosome (PHEX) [19,20]. These hormones interconnect the gastrointestinal-bone-renal axis to regulate phosphate homeostasis [21]. However, the changes observed in these phosphate-regulating peptides did not induce significant change in skeletal and serum phosphate levels in rats with MetS as compared to those without MetS [19,20]. The authors postulated that longer study duration might be required for the changes in phosphate-regulating peptides to be translated into observable change in bone phosphate level while circulating phosphate might be under tight hormonal control [20].

In humans, the relationship of phosphate metabolism and MetS appear to be inconclusive although majority of the studies found an inverse association (Table 1). A gender stratified analysis conducted in Japanese population with large sample size (*n* = 9076) demonstrated that decreased serum phosphate level was associated with increased incidence of MetS in male subjects. Specifically, it has been reported that waist circumference and FBG were higher, but HDL-C level was lower in individuals with low serum phosphate level [13]. Similar outcomes were obtained in a larger population (*n* = 46798) whereby lower phosphate level was correlated with MetS in subjects without a previous medical history [22]. Looking into the single component of MetS, there was a positive correlation between phosphate level with HDL-C meanwhile negative association was found between phosphate level with waist circumference, blood pressure, blood glucose, insulin, homeostatic model assessment for insulin resistance (HOMA-IR), and triglycerides (TG) [22,23]. Using case–control approach, serum phosphate concentration in MetS patients was lower in relative to subjects without MetS [14,24]. The lowering in serum phosphate concentration was proportional to the increasing in total number of MetS components [25,26,27].

Some studies demonstrated that higher serum phosphate concentration could be a risk factor for MetS. In Korean adolescents aged 12 to 18 years old, daily phosphorus intake was positively associated with systolic blood pressure, waist circumference, and daily calcium intake but negatively correlated with HDL-C [11]. Among the normal weight individuals, high phosphorus level was one of the factors associated with MetS [12]. The serum phosphate concentration was associated with occurrence of MetS in the group aged older than 60 years old, but no significant correlation was found between serum phosphate concentration and MetS in the younger groups. For each of the MetS component, the positive association between serum phosphate level with waist circumference, FBG, TG, and HDL-C remained significant [28]. On the other hand, prospective case–control study by Terzi et al. found no significant difference in the serum phosphorus level between postmenopausal women with and without MetS [15].

The postulated reasons for the differences concerning the relationship between serum phosphate level with MetS includes (a) different definition used for the diagnosis of MetS, (b) different cut-off point adopted for each of the MetS diagnostic criteria, (c) different subjects’ age and study population. In addition, bone health status and kidney function could be also the confounding factors for different phosphorus level/metabolism in MetS patients.

### 3.2. Phosphate Metabolism and Obesity

Overweight and obesity represent the emerging health burden worldwide, which are mainly attributed to imbalance in energy profile and physical inactivity. Current available evidence consistently reported an inverse relationship between obesity and phosphate level in children and adults (Table 2). Children with X-linked hypophosphatemia, an inherited disorder characterised by low circulating phosphate level, exhibited higher prevalence of overweight or obesity compared to the general population in a retrospective longitudinal observational study consisting of 172 boys and girls aged 5 to 20 years old [29]. In a case–control study, the recruited participants were divided into two categories: normal and obese children aged 6–12 years old as well as adolescents aged 12 to 16 years old. The findings indicated that serum phosphate concentration was lower in obese children than controls, but this association was not observed in adolescents [30]. The discrepancy in the research outcomes between children and adolescents might be due to the difference in metabolic rate and physical activity. Serum phosphate level was also found to be inversely associated with body mass index (BMI) in women [31,32]. A double-blind, randomised, placebo-controlled trial enrolled 63 overweight adults (18–45 years old) with normal kidney function to investigate the effects of phosphorus supplementation on body weight. The subjects were randomly assigned with placebo or phosphorus supplements (375 mg), which were taken together with three main meals (breakfast, lunch, and dinner) for a duration of 12 weeks. The results showed a significant lower body weight, BMI, waist circumference, and appetite scores in the phosphorus-supplemented group than the placebo group [33]. A randomised blinded cross-over study was conducted to assess the effects of phosphorus ingestion on energy metabolism in obese and lean subjects (*n* = 15) aged between 20 to 29 years old. The participants received either placebo or 500 mg phosphorus tablet with a high-carbohydrate meal (648 kcal) containing white bread, strawberry jam, butter, and orange juice. Measurement of energy metabolism was performed 30 min prior to and four hours after meal. The authors found that phosphorus supplementation with meal amplified postprandial energy expenditure in both obese and lean male subjects [34].

**Figure 1:**
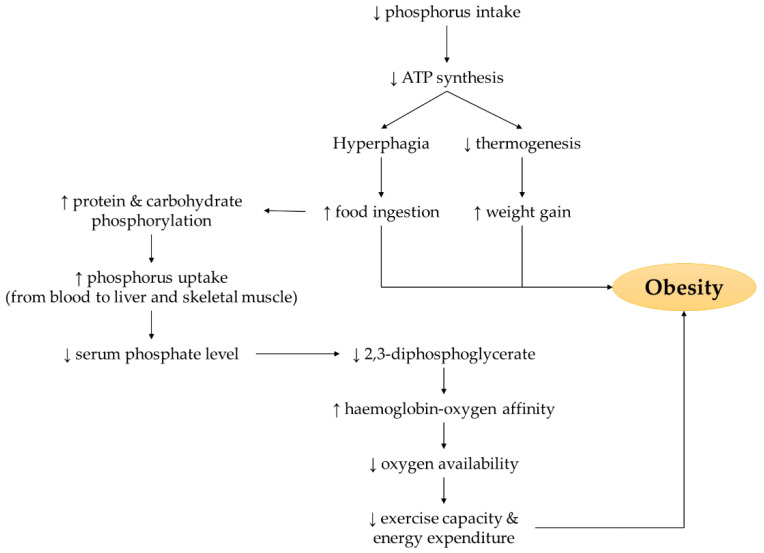
The mechanism of action underlying the possible link between phosphorus intake or circulating phosphate level with the occurrence of obesity. Note: The arrow pointing upward (↑) indicates an increase whereas the arrow pointing downward (↓) indicates a decrease.

The negative relationship between phosphorus status and weight gain is mediated through regulation of food intake, thermogenesis (a process of heat production), capacity of physical activity, and energy expenditure (Figure 1). Firstly, food ingestion promotes insulin release as well as requires proteins and carbohydrates phosphorylation, subsequently enhances phosphorus uptake from extracellular blood serum to liver and skeletal muscle that lowers serum phosphate level [35]. Secondly, low phosphorus intake limits phosphorus availability for adenosine triphosphate (ATP) synthesis. The signal of declined hepatic ATP production is transmitted to the central nervous system, leading to hyperphagia and increased food consumption [36]. Thirdly, low ATP production causes deficiency in thermogenesis and subsequently increases efficiency in weight gain [36]. Fourthly, low serum phosphorus is associated with reduced 2,3-diphosphoglycerate level, a compound that has a strong affinity towards deoxygenated haemoglobin. Reduced interaction between 2,3-diphosphoglycerate and haemoglobin increases the oxygen affinity towards haemoglobin, thus lowering the oxygen availability for oxidation, capacity for physical activity, and energy expenditure [36].

### 3.3. Phosphate Metabolism and Hyperglycaemia

Scientific evidence pointed out a link between phosphate metabolism and diabetes in vivo and in humans (Table 3). Male Sprague-Dawley rats fed with high phosphate diet had lower insulin level and HOMA-IR as compared to the rats fed with low phosphate diet [7]. Several human epidemiological studies revealed that serum phosphate level was negatively associated with serum postprandial glucose level and HOMA-IR, positively associated with insulin sensitivity, but not associated with insulin secretion in general population [31,32,37,38]. Phosphorus supplementation (500 mg) resulted in no significant change in insulin level in a pilot study recruiting healthy male subjects [39]. In a cross-over study recruiting 15 apparently healthy male subjects, glucose ingestion reduced serum phosphate level. The supplementation of phosphorus (500 mg) together with glucose solution improved postprandial blood glucose, insulin, and insulin sensitivity index. In this study, the increase in phosphate availability causes intracellular glucose phosphorylation and insulin release is highly dependent on circulating glucose concentration, thus resulting in decreased glucose and insulin levels. However, pre-ingestion of phosphorus 60 min prior to glucose load did not produce similar results, mainly because phosphorus is known to be absorbed in the body within an hour and a drop in postprandial phosphorus during glucose loading was expected [40]. The trend for negative correlation between HOMA-IR with serum phosphate level was also observed in obese children aged 6–12 years old [30]. However, the increase in calcium-phosphate product, but not serum phosphate level, was correlated with future development of diabetes in a longitudinal study with a duration of 5.2 years follow-up. [41]. These findings suggested the role of calcium in determining the risk of diabetes should not be neglected. In a large prospective cohort study, high dietary phosphorus (1477 ± 391 mg/day) exceeding the adult recommended daily intake of 550–700 mg/day was associated with increased risk of developing type 2 diabetes mellitus (T2DM) among French women [42].

**Figure 2:**
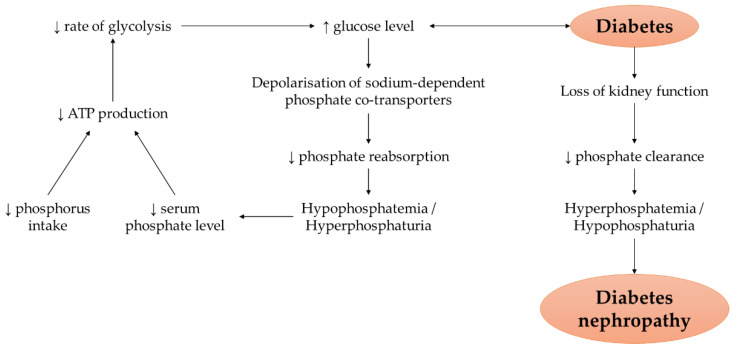
The mechanism of action underlying the possible link between phosphorus intake or circulating phosphate level with the occurrence of hyperglycaemia or diabetes and its associated nephropathy. Note: The arrow pointing upward (↑) indicates an increase whereas the arrow pointing downward (↓) indicates a decrease.

The close association between phosphate concentration/intake and metabolic control of glycaemic status is indisputable, mediated through the influence on the rate of cell glycolysis and phosphate handling in kidney tubules (Figure 2). Inorganic phosphate is an important component for ATP synthesis and ATP acts as a substrate for the activities of hexokinase and phosphofructokinase in glycolysis, facilitating the conversion from glucose to fructose-1,6-biphosphate. Hence, optimum replenish of ATP is crucial for a stable glucose metabolism [43]. Besides, glucose is a potent regulator of phosphate homeostasis in kidney energised by ATP. Elevated glucose concentration causes the depolarisation of sodium-dependent phosphate co-transporters at the proximal tubular cells, promoting the entry of inorganic phosphate and hyperphosphaturia. The restoration of blood glucose level results in improved phosphate reabsorption and subsequently increased serum phosphate concentration [44]. Nonetheless, hyperphosphatemia can develop in parallel with gradual loss of kidney function (a diabetes-related complication) attributed to declined phosphate clearance [45]. Two independent cohorts have demonstrated higher risk of chronic kidney disease in diabetic patients with high serum phosphate level and dietary phosphorus [46,47]. Indeed, both hypophosphatemia and hyperphosphatemia can occur in diabetes particularly hyperphosphatemia in diabetic nephropathy. It is recommended to maintain dietary and circulating phosphate levels at the normal range, whereby a lower or higher phosphate concentration than physiological range leads to the perturbation of glucose homeostasis and progression of diabetes.

**Table 3:** The relationship between phosphate metabolism with diabetes and its associated nephropathy.

Researcher (Year)	Study Type	Model/Subjects	Phosphorus/Phosphate-Related Parameters	Phosphorus/Phosphate-Related Outcomes	Reference
Abuduli et al. (2016)	Animal experimentation	Male Sprague-Dawley rats	Diet containing low (0.2%), normal (0.6%), or high (1.2%) phosphate	Rats fed with high phosphate diet had lower level of plasma insulin and HOMA-IR.	[7]
Akter et al. (2020)	Cross-sectional study	Apparently healthy adults (*n* = 1657, age: 18–78 years)	Phosphorus intake (514 ± 98 mg/day (quartile 1); 519 ± 110 mg/day (quartile 2); 514 ± 100 mg/day (quartile 3); 505 ± 106 mg/day (quartile 4))	Serum phosphate level and calcium-phosphate product were inversely correlated with HOMA-IR.	[38]
Haap et al. (2006)	Cross-sectional and longitudinal study	Healthy, non-diabetic adults (*n* = 881, age: 38 ± 1 years)	Serum phosphate level (3.4 ± 0.02 mg/dL)	Serum phosphate level was inversely correlated with 2-h glucose level, positively correlated with insulin sensitivity, but not correlated with insulin secretion. Serum phosphate level at baseline was correlated with higher postprandial glucose levels	[37]
Håglin et al. (2001)	Cross-sectional study	Men (*n* = 993, age: 50.8 ± 9.4 years) and women (*n* = 1272, age: 50.1 ± 10.7 years)	Serum phosphate level (0.98 ± 0.21 mmol/L (men); 1.06 ± 0.22 mmol/L (women))	Serum phosphate level was inversely correlated with blood glucose in men.	[32]
Håglin et al. (2014)	Cross-sectional study	Men and women (*n* = 2504, age: 50.4 ± 10.1 years)	Serum phosphate level (0.98 ± 0.20 mmol/L (men); 1.05 ± 0.21 mmol/L (women))	Serum phosphate level was inversely correlated with glucose level in men and women.	[31]
Hazim et al. (2014)	Pilot cross-over study	Healthy male subjects (*n* = 8, age: 19.25 ± 0.41 years)	Phosphorus supplementation (500 mg)	Phosphorus supplementation did not cause any change in insulin level.	[39]
Celik & Andiran (2011)	Case control study	Normal and obese children (*n* = 177, age: 6–12 years) and adolescents (*n* = 121, age: 12–16 years)	Serum phosphate level (4.8 ± 0.4 mg/dL (obese children); 5.1 ± 0.5 mg/dL (control–children); 4.4 ± 0.5 mg/dL (obese adolescents); 4.5 ± 0.6 mg/dL (control–adolescents))	Serum phosphate level was negatively associated with HOMA-IR in obese children.	[30]
Khattab et al. (2015)	Cross-over study	Healthy male subjects (*n* = 7, age: 23.22 ± 1.83 years)	Phosphorus supplementation (500 mg)–given together with glucose solution (75 g)	Co-ingestion of phosphorus and glucose improved postprandial glucose, insulin, and insulin sensitivity index	[40]
		Healthy male subjects (*n* = 8, age: 27.3 ± 1.68 years)	Phosphorus supplementation (500 mg)–given 60 min prior to glucose ingestion (75 g)	Pre-ingestion of phosphorus did not show similar effects.	
Lorenzo et al. (2014)	Longitudinal study	Non-diabetic adults (*n* = 863, age: 40–69 years)	Serum phosphate level (1.01 ± 0.001 mmol/L (non-diabetes); 1.04 ± 0.01 mmol/L (diabetes)) and calcium-phosphate product (2.32 ± 0.02 mmol^2^/L^2^ (non-diabetes); 2.41 ± 0.03 mmol^2^/L^2^ (diabetes))	No correlation between serum phosphate level and progression of diabetes. Increased calcium-phosphate product was associated with increased risk of T2DM	[41]
Mancini et al. (2018)	Prospective cohort study	French women (*n* = 71270)	Dietary phosphorus intake (1477 ± 391 mg/day)	High phosphorus intake was associated with risk of T2DM.	[42]
Xiang et al. (2018)	Cohort study	Patients with diabetic nephropathy (*n* = 591)	Serum phosphate level (1.0 ± 0.1 mmol/L (quartile 1); 1.2 ± 0.1 mmol/L (quartile 2); 1.3 ± 0.1 mmol/L (quartile 3); 1.5 ± 0.2 mmol/L (quartile 4))	High serum phosphate level was associated with increased risk of diabetic nephropathy (HR = 2.88; 95% CI 1.12–5.04).	[46]
Yoon et al. (2017)	Prospective community-based cohort study	Subjects with diabetes mellitus (*n* = 873; aged 55.6 ± 8.7 years) and without diabetes (*n* = 5846; aged 51.4 ± 8.6 years)	Dietary phosphorus intake (< 0.46 mg/kcal (quartile 1); 0.46 to <0.50 mg/kcal (quartile 2); 0.50 to <0.55 mg/kcal (quartile 3); ≥0.55 mg/kcal (quartile 4))	High dietary phosphorus was associated with risk of chronic kidney disease development in patients with diabetes mellitus.	[47]

Abbreviation: HOMA-IR, homeostatic model assessment for insulin resistance; T2DM, type 2 diabetes mellitus.

### 3.4. Phosphate Metabolism and Hypertension

The relationship between phosphate metabolism and hypertension is of important as processed food contains large amount of inorganic phosphate-based food additives, which is readily absorbed by gastrointestinal tract [48]. Animal experimental studies highlighted the detrimental role of phosphorus excess in raising blood pressure (Table 4). The feeding of high phosphate and zinc-free diet increased systolic blood pressure with reduced left ventricular systolic and diastolic function in the spontaneous hypertensive rats and control rats [49]. Using male Sprague-Dawley rats, resting blood pressure was increased after feeding with a high phosphorus diet [50,51], which was returned to normal after the addition of lanthanum carbonate (a phosphate binder) into the diet [51].

However, the preclinical findings did not translate into clinical settings whereby inconsistent findings exist for the association between phosphate intake and blood pressure. Subjects with higher phosphorus intake through diet or supplement use had lower systolic and diastolic blood pressures in adults aged 40 years and above [52,53]. Longitudinal study also demonstrated that phosphorus intake from dairy products was associated with reduced incidence of hypertension in subjects free of cardiovascular disease after an average of 6.2 years follow-up [52]. Study by McClure et al. recruited individuals with non-optimal systolic and diastolic blood pressure aged 25 to 79 years old. Consumption of added phosphorus (in the form of phosphates and phosphoric acids), but not phosphorus-containing food derived from plants and animals, resulted in increases in systolic and diastolic blood pressure. They also revealed direct longitudinal association between urinary phosphorus excretion and diastolic blood pressure. However, total phosphorus intake was not associated with changes in blood pressure [54]. Similar outcomes were observed in healthy African American adults, whereby no significant association was found between phosphorus intake with systolic blood pressure [55]. The addition of sodium phosphate into the diet resulted in elevation of systolic and diastolic blood pressure in healthy young adults in two independent human studies [56,57].

**Table 4:** The relationship between phosphate metabolism and hypertension.

Researcher (Year)	Study Type	Model/Subjects	Phosphorus/Phosphate-Related Parameters	Phosphorus/Phosphate-Related Outcomes	Reference
Suzuki et al. (2014)	Animal experimentation	Male spontaneous hypertensive rats and Wistar Kyoto rats	Control diet (0.3% phosphorus) or high phosphorus (1.2%) and zinc-free diet	High phosphorus and zinc-free diet raised systolic blood pressure in both rat models.	[49]
Bozic et al. (2014)	Animal experimentation	Male Sprague-Dawley rats	Moderate-phosphate (0.6%) or high-phosphate (1.2%) diet	High phosphate diet increased blood pressure, plasma renin activity, angiotensin II, left ventricular hypertrophy, and PTH in rats. The addition of lanthanum carbonate, a phosphate binder reversed these changes.	[51]
Mizuno et al. (2016)	Animal experimentation	Male Sprague-Dawley rats	Normal phosphate (0.6%) or high phosphorus (1.2%) diet	High phosphorus diet increased blood pressure in rats. High phosphorus diet activated skeletal muscle exercise pressor reflex function, resulting in greater increases sympathetic nerve activity.	[50]
Elliott et al. (2008)	Cross-sectional study	Adult men and women (*n* = 4680, age: 40–59 years)	Phosphorus intake (439–662 mg/day)	Phosphorus intake was inversely correlated with blood pressure.	[53]
Alonso et al. (2010)	Cohort study	Adult men and women free of cardiovascular diseases (*n* = 13,444, age: 45–84 years)	Phosphorus intake (787 mg/day (quintile 1); 968 mg/day (quintile 2); 1063 mg/day (quintile 3); 1171 mg/day (quintile 4); 1526 mg/day (quintile 5))	Subjects in the higher quintile of phosphorus intake at baseline had lower baseline systolic and diastolic blood pressure.	[52]
	Longitudinal study	Adult men and women free of cardiovascular diseases (*n* = 11,109, age: 45–84 years)		Phosphorus intake from dairy products was associated with lower blood pressure and reduced risk of hypertension.	
McClure et al. (2020)	Longitudinal study	Adult men and women with non-optimal blood pressure (*n* = 806, age: 25–79 years)	Phosphorus intake (1154 ± 408 mg/day) and excretion (937 ± 384 mg/day)	No association between phosphorus intake and blood pressure. Increased urinary phosphorus excretion was associated with an increase in diastolic blood pressure. Added phosphorus (not derived from plant or animal sources) was associated with the increases in systolic and diastolic blood pressure.	[54]
Olivo et al. (2019)	Cross-sectional study	African American adults (*n* = 973, age: 59.3 ± 10.8 years)	Phosphorus intake (231–801 mg/day (quartile 1); 802–1055 mg/day (quartile 2); 1056–1420 mg/day (quartile 3); 1421–3769 mg/day (quartile 4))	No association between phosphorus intake and systolic blood pressure.	[55]
Mohammad et al. (2018)	Prospective study	Young adults (low phosphate group (*n* = 10, age: 23.4 ± 3.4 years); high phosphate group (*n* = 10, age: 23.1 ± 3.2 years))	Low phosphate (0.7 mmol/kg sodium chloride) or high phosphate (1 mmol/kg/day sodium phosphate) diet	High phosphate diet increased 24-h systolic blood pressure, diastolic blood pressure, pulse rate, urinary metanephrine and normetanephrine excretion.	[56]
Zhang et al. (2021)	Open-label prospective cross-over study	Young healthy male volunteers with normal nutritional status and without any medication use (*n* = 6, age: 29 ± 2 years)	Low (500 mg/day), normal (1500 mg/day), or high (2300 mg/day) phosphorus diet	High phosphorus diet increased systolic blood pressure. High phosphorus diet increased serum PTH, FGF-23, and atrial natriuretic peptide but reduced 1,25(OH)_2_D, aldosterone, and 24-h urine volume.	[57]
Håglin et al. (2001)	Cross-sectional study	Men (*n* = 993, age: 50.8 ± 9.4 years) and women (*n* = 1272, age: 50.1 ± 10.7 years)	Serum phosphate level (0.98 ± 0.21 mmol/L (men); 1.06 ± 0.22 mmol/L (women))	Serum phosphate level was inversely correlated with blood pressure in men and women.	[32]
Kesteloot & Joossens (1988)	Epidemiological survey	Men and women (*n* = 8058, mean age: 49 years)	Serum phosphate level (1.05 ± 0.17 mmol/L (men); 1.08 ± 0.16 mmol/L (women))	Serum phosphate level was inversely correlated with systolic blood pressure	[58]
Huang et al. (2008)	Cross-sectional and longitudinal study	Haemodialysis patients (*n* = 707, age: ≥17 years)	Serum phosphate level (<4.42 mg/dL (quintile 1); 4.42–5.21 mg/dL (quintile 2); 5.21–6.07 mg/dL (quintile 3); >6.07 mg/dL (quintile 4))	Serum phosphate level was positively correlated with systolic blood pressure and pulse pressure at baseline and subsequent follow-up at 3, 6, 12, 18, and 27 months.	[59]
Patel et al. (2015)	Longitudinal study	Hypertensive adults (*n* = 9260, age: 51.7 ± 14.6 years)	Serum phosphate level (1.0 ± 0.2 mmol/L (men); 1.1 ± 0.2 mmol/L (women))	Higher serum phosphate level was correlated with poor systolic blood pressure reduction as well as all-cause and cardiovascular mortality in hypertensive adults. Higher serum phosphate level was correlated with poor survival in hypertensive adults with chronic kidney disease.	[60]
Kanbay et al. (2007)	Cross-sectional study	Dipper (*n* = 76, age: 51.4 ± 13.4 years) and non-dippers (*n* = 114, age: 53.4 ± 12.8 years) hypertensive patients	Serum phosphate level (3.3 ± 0.4 mg/dL (dippers); 3.6 ± 0.5 mg/dL (non-dippers))	Non-dipper patients had higher levels of phosphate, calcium-phosphate product, and PTH. Serum phosphate and PTH levels were predictors for non-dipper hypertension.	[61]

Abbreviation: 1,25(OH)_2_D, 1,25-dihydroxycholecalciferol/calcitriol; FGF-23, fibroblast growth factor-23; PTH, parathyroid hormone.

The discrepancy on the outcomes might be attributable to several reasons. The difference in experimental designs adopted, whether through validated food frequency questionnaires, 24-h food recall, or phosphorus-supplemented diet, might cause variability in capturing the precise amount of phosphate intake. Besides, the detail on phosphorus content is not compulsory in the nutrition fact by food manufacturer thus affecting the phosphate intake estimation. The effects of phosphate intake on blood pressure may differ depending on the source of dietary phosphate. Phosphate derived from natural food source (such as nuts, grains, seeds, fruits, vegetable, meat, fish, poultry, dairy products, and eggs) might be beneficial or had negligible effect on blood pressure level whereas phosphate derived from additives (commonly used as flavour enhancer and preservatives in processed foods) can be deleterious. Phosphorus from plants exists in the form of phytic acid, which requires phytase enzyme to release its phosphate content; thus, the oral bioavailability of plant-derived phosphate is low as compared to inorganic phosphate.

Researchers have investigated the relationship between serum phosphate level with blood pressure and risk of hypertension in humans. In healthy individuals, there was a negative correlation between serum phosphate level and systolic blood pressure in men and women in earlier studies [32,58]. On the other hand, positive association was seen between serum phosphate concentration and blood pressure under pathological conditions. In a study consisting of incident haemodialysis patients, cross-sectional analysis showed that higher serum phosphate was correlated with higher predialysis systolic blood pressure and pulse pressure at baseline and subsequent follow-up until 27 months [59]. Hypertensive adults with elevated serum phosphate were closely linked with poor systolic blood pressure reduction, cardiovascular mortality, and poor survival (particularly in those with chronic kidney disease) in a 5-year follow-up [60]. Serum phosphate concentration has been identified as the predictors for non-dipper hypertensive patients [61].

**Figure 3:**
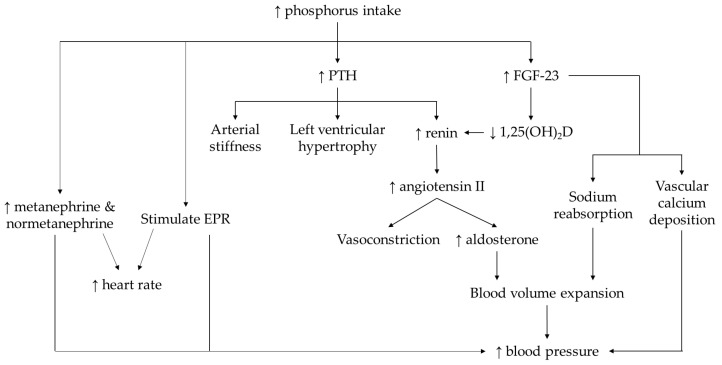
The mechanism of action underlying the possible link between phosphorus intake or circulating phosphate level with the occurrence of hypertension. Note: The arrow pointing upward (↑) indicates an increase whereas the arrow pointing downward (↓) indicates a decrease.

High phosphate diet induces overstimulation of sympathetic nervous system, modulates renin-angiotensin-aldosterone system (RAAS), altered phosphorus-regulating hormones, increased vascular thickness, and impaired endothelium-dependent vasodilation, contributing to its blood pressure-raising effect (Figure 3). Direct evidence showed that chronic exposure of a high phosphate diet stimulated exercise pressor reflex in skeletal muscle, augmented resting blood pressure and heart rate in normotensive conditions with the absence of renal failure [50]. Another study showed that the increase in 24-h ambulatory blood pressure after feeding on a high phosphate diet occurred along with tachycardia, excretion of metanephrine and normetanephrine [56]. These findings suggested sympathetic nerve overstimulation upon ingestion of high phosphate diet. The RAAS is a critical regulator for blood pressure by controlling blood volume, electrolyte balance, and vascular resistance. Animals fed with a high phosphate diet elevated renin expression in the kidney, plasma renin activity, and angiotensin II level [51]. In healthy individuals exposed to high phosphorus diet, the elevation of systolic blood pressure was due to volume expansion (evidenced by the decrease in urine volume). In response to volume expansion, the secretion of atrial natriuretic peptide was increased while aldosterone level was decreased to promote urinary excretion [57].

Phosphate loading is often associated with the changes in PTH, FGF-23, and vitamin D. High phosphorus diet increased serum PTH and FGF-23 but reduced 1,25-dihydroxycholecalciferol (1,25(OH)_2_D) levels [51,57]. The dysregulation of RAAS has been suggested as the molecular link between these hormonal changes and hypertension. The mechanistic actions of PTH to hypertension include the increase in renin secretion as well as its direct effects on arteries and myocytes to promote arterial stiffness and left ventricular hypertrophy [51,62]. The upregulation of FGF-23 contributes to vascular calcium deposition and sodium reabsorption in sodium chloride cotransporter, resulting in blood volume expansion and hypertension [62]. FGF-23 also inhibits the activation of 1,25-dihydroxyvitamin D, a potent suppressor for renin synthesis [63]. Hyperparathyroidism, raised FGF-23, vitamin D deficiency can be the results of excessive inorganic phosphate intake, which were closely associated with increased risk of hypertension.

### 3.5. Phosphate Metabolism and Dyslipidaemia

Dyslipidaemia is defined as imbalanced circulating lipids (including TG, total cholesterol (TC), low-density lipoprotein cholesterol (LDL-C), and HDL-C) that lead to the development of cardiovascular diseases. Preclinical experimentations unambiguously reported the benefits of phosphate intake on cholesterol metabolism (Table 5). Tanaka and colleagues conducted an experiment to investigate the effects of dietary phosphate restriction on hepatic lipid accumulation and lipid metabolism using a mouse model. Mice on inorganic phosphate-restricted diet (0.1%) had higher liver weight and hepatic lipid accumulation as compared to those on inorganic phosphate-sufficient diet (1.2%). Plasma phosphate level was also negatively correlated with TC in both phosphate-restricted and phosphate-sufficient groups [64]. The same group of researchers investigated the relationship between phosphate and cholesterol metabolism using a different approach. Mice with sodium-dependent phosphate co-transporter (Npt2a) deficiency was used as a model of hypophosphatemia and fed with diet with or without 2% cholesterol. The Npt2a-deficient mice exhibited higher plasma lipid levels than the wild type mice [65]. Similar outcomes were obtained using a low-density lipoprotein receptor knockout (Ldlr^-/-^) mouse model. The feeding of high and adequate dietary phosphorus markedly reduced TG and cholesterol in serum as well as increased faecal lipid excretion [66]. Male Sprague-Dawley rats fed with high phosphate diet (1.2%) for four weeks had lower visceral fat accumulation and non-esterified fatty acids [7].

In human, the relationship between phosphate metabolism and dyslipidaemia remained inconclusive. Using a placebo-controlled, double-blind, cross-over study design, the effects of pentacalcium hydroxy-triphosphate supplementation through incorporation into bread on lipid profile was assessed in young healthy volunteers. The serum concentrations of TC, LDL-C, and ratio of LDL-C:HDL-C were lowered in participants provided with pentacalcium hydroxy-triphosphate-incorporated bread. Bile acid and cholesterol excretion were also increased [67]. A pilot cross-over trial recruiting eight healthy male subjects indicated no difference in non-esterified fatty acids and TG between subjects receiving high-fat meal with placebo and phosphorus (500 mg) [39]. However, the relatively small sample size in these studies might not provide better representation on the relationship between phosphate level and dyslipidaemia in a population. Using a larger sample size, a cross-sectional study consisting of 2504 men and women demonstrated a positive relationship between serum phosphate and TC levels in non-type 2 diabetes subjects [31]. Herein, the research gaps remain to be investigated are validation on the inconclusive association between serum phosphate concentration and lipid profile as well as the effects of phosphate supplementation on lipid profile in hypercholesterolemic subjects.

**Figure 4:**
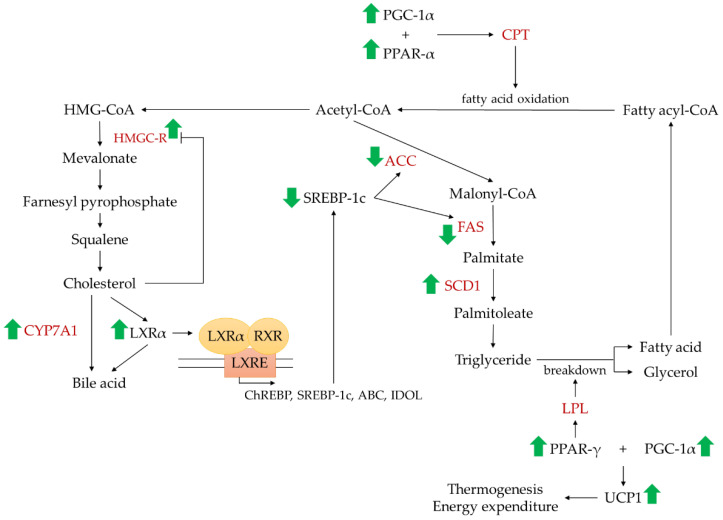
The mechanism of action underlying the possible link between phosphorus intake or circulating phosphate level with the occurrence of dyslipidaemia. The green arrows pointing up (↑) indicate upregulation whereas pointing down (↓) indicates downregulation of genes by phosphorus. Abbreviation: ABC, ATP binding cassette; ACC, acetyl-CoA carboxylase; ChREBP, carbohydrate-response element-binding protein; CPT, carnitine palmitoyl transferase; CYP7A1, cholesterol 7 alpha-hydroxylase; FAS, fatty acid synthase; HMG-CoA, 3-hydroxyl-3-methylglutaryl-coenzyme A; HMGC-R, 3-hydroxyl-3-methylglutaryl-coenzyme A reductase; IDOL, inducible degrader of the LDL-receptor; LPL, lipoprotein lipase; LXRα, liver X receptor-alpha; LXRE, LXRα-responsive elements; PGC-1α, peroxisome proliferator-activated receptor-γ coactivator-1α; PPAR-α, peroxisome proliferator-activated receptor-alpha; PPAR-γ, peroxisome proliferator-activated receptor-gamma; RXR, retinoid X receptor; SCD1, stearoyl-CoA desaturase-1; SREBP-1c, sterol regulatory element-binding protein-1c; UCP1, uncoupling protein 1.

The mechanism of actions underlying the effects of phosphate in cholesterol metabolism have been elucidated in preclinical settings (Figure 4). The sufficiency of phosphate content in diet resulted in lower hepatic lipid accumulation along with higher expression of 3-hydroxyl-3-methylglutaryl-coenzyme A reductase (HMGC-R) in mice [64]. The cholesterol biosynthesis is mediated through mevalonate pathway, which begins from simple precursor such as acetyl coenzyme A (acetyl-CoA) that undergoes a series of enzymatic reactions mainly regulated by HMGC-R to promote the conversion of 3-hydroxyl-3-methylglutaryl-coenzyme A (HMG-CoA) into mevalonate [68]. HMGC-R activity are mediated through negative feedback regulation by free cholesterol. Besides, cholesterol induces HMGC-R ubiquitination and promotes its degradation [69]. Thus, lower dietary/circulating cholesterol is often associated with higher expression of HMGC-R [70]. Adequate phosphate intake was also associated with suppressed fatty acid synthase (FAS) and acetyl-CoA carboxylase (ACC) but elevated stearoyl-CoA desaturase-1 (SCD1) expression [7,64]. The lipogenic pathway in liver is initiated with the carboxylation of acetyl-CoA by ACC to produce malonyl-CoA. It is then utilised by FAS to produce long chain saturated fatty acid (palmitate) and eventually the conversion to monounsaturated fatty acid (palmitoleate) catalysed by SCD1 via forming a double bond [71]. Monounsaturated fatty acids are the substrates for the synthesis of membrane phospholipids and TG [72].

Dietary phosphate also influenced the expression of transcription factors responsible for cholesterol metabolism and fatty acid biosynthesis, evidenced by the rising of liver X receptor-alpha (LXRα), peroxisome proliferator-activated receptors (PPAR)-α, PPAR-γ, and peroxisome proliferator-activated receptor-γ coactivator-1α (PGC-1α) but lowering of sterol regulatory element-binding protein (SREBP)-1c in animals as compared to the group fed on phosphate-deficient diet [7,64]. LXR is a transcription factor belongs to nuclear receptor superfamily, acting as a master regulator in cholesterol metabolism, inflammatory signalling, and immune response. Various cholesterol derivatives, including oxidised forms of cholesterol and cholesterol precursors, are natural ligands that activate LXRα. Upon activation, LXRα heterodimerise with retinoid X receptor (RXR) and bind to LXRα-responsive elements (LXREs). Subsequent transcription of genes ensues, including SREBP-1c, carbohydrate-response element-binding protein (ChREBP), ATP binding cassette (ABC), and inducible degrader of the LDL-receptor (IDOL), responsible for the regulation of lipogenic pathway [73]. SREBP-1c induces lipogenesis by upregulating FAS and ACC as well as promotes the storage of excess fatty acid as TG [74]. Peroxisome proliferator-activated receptors (PPARs) belongs to the nuclear hormone receptor superfamily of ligand-activated transcription factors. It exists in three subtypes (PPAR-α, PPAR-γ, and PPAR-β/δ), which differ in their function [75,76]. PPAR-α responds to free fatty acid concentration and promotes the expression of gene involved in fatty acid oxidation, carnitine palmitoyl transferase (CPT) [77]. Meanwhile, PPAR-γ favours the expression of lipoprotein lipase (LPL) that facilitates the hydrolysis of TG into glycerol and two free fatty acids [78]. PGC-1α is a transcription factor that binds to PPAR-α, PPAR-γ, and PPAR-β/δ facilitating fatty acid oxidation and utilization.

Apart from lipogenic genes and transcription factors, phosphorus altered the levels of apolipoproteins and the receptor mediating the cellular uptake of lipoproteins. Phosphorus supplementation increased postprandial apolipoprotein B48 (apoB48) but decreased apolipoprotein B100 (apoB100) in these healthy male subjects in a pilot crossover trial [39]. The small sample size used in this study might not reflect stronger statistical and clinical implications. In addition, similar study design should be adopted to investigate the effects of phosphorus ingestion on apoB in women [39]. In mice, the feeding of phosphate-sufficient diet raised the expression LDL-receptor (LDL-R) gene [64]. Recent study demonstrated that reduced hepatic sterol exporters and lipoprotein receptors were detected in mice provided with adequate and high phosphorus diet [66]. Apolipoprotein B (apoB) is a structural protein that found on chylomicron and several types of lipoproteins. It appears naturally in two main isoforms, apoB48 and apoB100 [79]. ApoB48 is the specific markers for intestinal chylomicron particles whereas apoB100 is an integral component of very low-density lipoprotein cholesterol (VLDL-C), IDL, and LDL particles. Hence, they can be useful markers for the estimation of chylomicrons and VLDL-C production respectively [80,81]. The absorption of digested dietary lipids in the form of chylomicrons that contains apoB48 and increase in formation of VLDL-C that contain apoB100 are the characteristics of postprandial hyperlipidaemia [39]. LDL-R is a cell surface receptor that recognises apoB100 and apolipoprotein E (apoE), thus mediating the cellular uptake of cholesterol-rich lipoprotein particles via endocytosis [82].

Scientific evidence indicated that adequate phosphate consumption increased cholesterol 7 alpha-hydroxylase (CYP7A1) in mice [64]. CYP7A1 is an enzyme catalysing the conversion of cholesterol to 7-alpha-hydroxycholesterol, a crucial step in bile acid synthesis. The inhibition of CYP7A1 represses bile acid biosynthesis. In the state of high cholesterol level, CYP7A1 is upregulated by LXR to increase the production of bile acids and reduced hepatic cholesterol level. When cholesterol level is low, CYP7A1 is downregulated by SREBP. Nonetheless, brown adipose tissue plays an important role in lipid oxidation by regulating thermogenesis. The high phosphate diet group showed increased uncoupling protein 1 (UCP1), the major uncoupling protein isoform expressed in brown adipose tissue, as compared to the control diet group [7]. Upregulated UCP1 is often associated with increased thermogenesis and energy expenditure to protect from obesity and fat accumulation.

## 4. Perspectives

Most of the studies investigated the effects phosphorus supplementation on MetS components using doses that fall within the daily recommended intake (≤700 mg/day) with exception of few studies investigated on high phosphorus intake (>700 mg/day). The participants recruited in the studies included had a tightly regulated serum phosphate level within normal range. Phosphorus/phosphate appears as a double edge sword that has positive and negative effects on the metabolic processes during MetS condition. Based on the scientific evidence, most of the studies found that higher serum phosphate level prevented obesity, improved postprandial blood glucose level, lowered insulin resistance, and increased insulin sensitivity. Nonetheless, higher phosphate intake exceeding the recommended dietary allowance was potentially associated with increased risk of T2DM in healthy population. The in vivo studies revealed the blood pressure-rising effects of high phosphate diet. Human studies demonstrated that the effects of phosphorus intake on hypertension remained inconclusive, highly depending on the source of phosphorus and study approach. Adequate phosphate level in serum was beneficial on blood pressure in healthy individuals but detrimental to subjects with pathological conditions. For lipid metabolism, preclinical studies supported the positive effects of sufficient or high phosphate diet in maintaining a well-balanced lipid profile. In human, phosphate ingestion might be advantageous to healthy subjects, but high circulating phosphate level might result in high cholesterol under diabetic conditions. In MetS, three or more of these features are often co-exist in the same individual. It is postulated that phosphate acts both positively and negatively in the progression of MetS. The net outcome may differ from one individual to another, depending on the collective features that define MetS.

Several considerations need to be acknowledged in current evidence. The causal relationship (whether MetS causes phosphate disturbance or phosphate intake/level affects development of MetS) could not be determined for human studies conducted in cross-sectional approach. Only a small number of studies was conducted longitudinally; thus, more studies are recommended to confirm whether the relationship between phosphate and MetS to be causal in nature. The study subjects included are specific to certain population, medical condition, and/or healthy volunteer; therefore, caution should be exercised when generalising to other populations. The levels of vitamin D, PTH, molecules that regulates phosphate reabsorption (FGF-23 and PHEX), as well as 2,3-diphosphoglycerate were not assessed in some of these studies. These measurements should not be neglected in future studies with the purpose of investigating the role of phosphate metabolism in MetS. Some studies were conducted in small sample size; thus, the outcome obtained may not represent the findings of a large population. In addition, the potential of serum phosphate concentration as a biomarker to represent overall dietary phosphate intake and determine the occurrence of MetS need to be further validated as it is also influenced by renal function. In this context, the effects of phosphate metabolism on MetS should be compared in patients with normal and impaired kidney function. The idea of different phosphate source (either from plants, animals, or additives) exerts different impact on blood pressure has been suggested by available evidence. However, the investigation of phosphate derived from different sources on other MetS components including obesity, blood sugar, insulin, and lipid metabolism is limited, hence serving as the potential research gap to be filled by researchers. The source of the phosphorus supplement provided to the subjects should be mentioned. We also addressed the limitations of current review. This review aims to provide an overview understanding on the effects of phosphate alone in influencing MetS and its individual elements. Vitamin D, which are strongly connected to phosphate metabolism, was not discussed further in this review.

## 5. Conclusions

The phosphorus intake and level of serum phosphate could be an important factor in the pathogenesis of MetS. Phosphate intake level lower than or exceeding the recommended range may predispose an individual to be at risk of MetS. Hence, it is recommended that the dietary and circulating phosphate level should be measured as part of the management for MetS. Future studies investigating the causal relationship between phosphate levels and prevalence of MetS are recommended.

## Figures and Tables

**Table 1:** The relationship between phosphate metabolism and MetS.

Researcher (Year)	Study Type	Model/Subjects	Definition of MetS	Phosphorus/Phosphate-Related Parameters	Phosphorus/Phosphate-Related Outcomes	Reference
Shimodaira et al. (2017)	Cross-sectional study	Japanese men (*n* = 9076) and women (*n* = 6965; age: ≤80 years)	Fulfilment of ≥3 components: Waist circumference (men: ≥90 cm; women: ≥80 cm) Blood pressure: ≥130/85 mm Hg or receiving treatment for hypertension TG ≥ 150 mg/dL or receiving treatment for hypertriglyceridemia HDL-C (men: <40 mg/dL; women: <50 mg/dL) or receiving treatment for low HDL-C FBG ≥ 100 mg/dL or previously diagnosed diabetes	Serum phosphate level (3.04 ± 0.44 mg/dL (men with MetS); 3.13 ± 0.42 mg/dL men with non-MetS); 3.53 ± 0.41 mg/dL (women with MetS); 3.54 ± 0.41 mg/dL (women with non-MetS))	Serum phosphate level was lower in men with MetS than those without MetS, but not in women. Serum phosphorus level was positively associated with HDL-C, but negatively associated with waist circumference and FBG in both men and women. Lower serum phosphorus level was associated with higher prevalence of MetS in men, but not in women.	[13]
Park et al. (2009)	Cross-sectional study	Subjects without a previous medical history (*n* = 46798, age: ≥20 years	Fulfilment of ≥3 components: Waist circumference (men: ≥90 cm; women: ≥80 cm) Blood pressure: ≥130/85 mm Hg TG ≥ 150 mg/dL HDL-C (men: <40 mg/dL; women: <50 mg/dL) FBG ≥ 110 mg/dL	Serum phosphate level (3.41 ± 0.43 mg/dL (men); 3.64 ± 0.42 mg/dL (women))	Serum phosphate level was positively associated with total cholesterol (TC), HDL-C, lipoprotein A, apolipoprotein A1, and calcium; but negatively associated with body mass index (BMI), waist circumference, FBG, insulin, HOMA-IR, high sensitivity C-reactive protein (hs-CRP), TG, systolic blood pressure, and diastolic blood pressure.	[22]
Grima et al. (2012)	Prospective, cross-sectional, single-centre study	Human immunodeficiency virus-1 (HIV-1)-infected patients (*n* = 121, age: >18 years)	Fulfilment of ≥3 components: Waist circumference (men: ≥102 cm; women: ≥88 cm) Blood pressure: ≥130/85 mm Hg TG ≥ 150 mg/dL HDL-C (men: <40 mg/dL; women: <50 mg/dL) FBG ≥ 110 mg/dL	Serum phosphate level (3.5 ± 0.6 mg/dL (control); 3.1 ± 0.6 mg/dL (MetS))	Serum phosphate level was inversely correlated with blood pressure, glucose, waist circumference, insulin, and TG, but directly correlated with HDL-C.	[23]
Ghanei et al. (2015)	Case control study	Patients with MetS (*n* = 122, age: 46.3 ± 11.8 years) and without MetS (*n* = 128, age: 45.3 ± 12.3 years)	Fulfilment of ≥3 components: Waist circumference (men: ≥102 cm; women: ≥88 cm) Blood pressure: ≥130/85 mm Hg TG ≥ 150 mg/dL HDL-C (men: <40 mg/dL; women: <50 mg/dL) FBG ≥ 100 mg/dL	Phosphorus intake (1336.0 ± 485.5 mg/day (control); 1439 ± 372.1 mg/day (MetS)) and serum phosphate level (3.9 ± 0.3 mg/dL (control); 3.3 ± 0.2 mg/dL (MetS))	Serum phosphate level was lower in subjects with MetS.	[24]
Stoian & Stoica (2014)	Case-control study	Subjects with (*n* = 64, age: 48.8 years) and without MetS (*n* = 91, age: 48.7 years)	Fulfilment of ≥3 components: Waist circumference (men: ≥102 cm; women: ≥88 cm) Blood pressure: ≥130/85 mm Hg TG ≥ 150 mg/dL HDL-C (men: <40 mg/dL; women: <50 mg/dL) FBG ≥ 100 mg/dL	Serum phosphate level (3.3 ± 0.5 mg/dL (control); 3.0 ± 0.5 mg/dL (MetS))	Subjects with MetS had lower serum phosphate level as compared to controls.	[14]
Kalaitzidis et al. (2005)	Case control study	Individuals with MetS (*n* = 64, age: 48.8 ± 11.1 years) and controls (*n* = 191, age: 48.7 ± 9.9 years)	Fulfilment of ≥3 components: Waist circumference (men: ≥102 cm; women: ≥88 cm) Blood pressure: ≥130/85 mm Hg TG ≥ 150 mg/dL HDL-C (men: <40 mg/dL; women: <50 mg/dL) FBG ≥ 110 mg/dL	Serum phosphate level (3.3 ± 0.5 mg/dL (control); 3.0 ± 0.5 mg/dL (MetS))	Serum phosphate level was lower in subjects with MetS than controls.	[25]
Gudmundsdottir et al. (2008)	Longitudinal study	Caucasian middle-aged men (*n* = 56, age: 42.1 ± 0.5 years)	Characteristics of MetS subjects: Waist circumference ≥94 cm Blood pressure: ≥140/90 mm Hg TG ≥ 150 mg/dL or receiving treatment for hypertriglyceridemia HDL-C < 40 mg/dL or receiving treatment for low HDL-C FBG ≥ 100 mg/dL HOMA-IR ≥ 2.8 Uric acid ≥ 363 μmol/L Homocysteine ≥ 11.6 μmol/L Fibrinogen ≥ 3.2 g/L	Serum phosphate level (1.02 ± 0.13 mmol/L (normotensive); 0.86 ± 0.13 mmol/L (hypertensive))	Serum phosphate level was inversely correlated with mean blood pressure. Individuals with lowest serum phosphate level had the highest number of MetS risk factors.	[27]
Vyssoulis et al. (2010)	Cohort study	White-coat hypertensive patients (*n* = 2600, age: ≥18 years)	Fulfilment of hypertension (blood pressure: ≥140/90 mm Hg) and at least 2 other components: Waist circumference (men: ≥102 cm; women: ≥88 cm) TG ≥ 150 mg/dL HDL-C (men: <40 mg/dL; women: <50 mg/dL) FBG ≥ 110 mg/dL	Serum phosphate level (3.53 ± 0.36 mg/dL (1 MetS component); 3.50 ± 0.38 mg/dL (2 MetS components); 3.49 ± 0.38 mg/dL (3 MetS components); 3.44 ± 0.36 mg/dL (4 MetS components); 3.35 ± 0.31 mg/dL (5 MetS components))	Patients with low serum phosphate level had higher incidence of non-dipping nocturnal systolic blood pressure. Serum phosphate level were higher in patients with lesser MetS components than those with more MetS features.	[26]
Park & Han (2021)	Cross-sectional study	Korean adolescents (*n* = 895, age: 12–18 years)	Fulfilment of ≥3 components: Waist circumference (men: ≥90 cm; women: ≥85 cm) Blood pressure: ≥130/85 mm Hg TG ≥ 150 mg/dL HDL-C (men: <40 mg/dL; women: <50 mg/dL) FBG ≥ 100 mg/dL	Phosphorus intake (1271.34 ± 594.53 mg/day (male); 938.41 ± 418.75 mg/day (female))	Daily phosphorus intake was correlated with systolic blood pressure (*r* = 0.448, *p* < 0.001), waist circumference (*r* = 0.115, *p* = 0.001), HDL-C (*r* = −0.113, *p* = 0.002), and daily calcium intake (*r* = 0.697, *p* < 0.001) Excessive phosphorus intake increased risk of MetS in adolescents.	[11]
Osadnik et al. (2020)	Cross-sectional study	Normal weight adults (*n* = 460, age: 18–35 years)	Fulfilment of ≥2 components: Blood pressure: ≥130/85 mm Hg TG > 150 mg/dL HDL-C (men: <1 mmol/L; women: <1.2 mmol/L) TC > 5.2 mmol/L FBG > 5.55 mmol/L	Serum phosphorus level (1.14 ± 0.15 mmol/L (non-MetS); 1.06 ± 0.18 mmol/L (MetS))	Serum phosphorus level was associated with MetS in normal weight individuals (odd ratio (OR) = −0.82; 95% confidence interval (CI) 0.67–0.99).	[12]
Jhuang et al. (2019)	Cohort study	Taiwan population (*n* = 1491, age: 29.26 ± 5.57 years (group 1); 49.21 ± 5.64 years (group 2); 69.21 ± 8.05 years (group 3))	Fulfilment of central obesity (waist circumference, men: ≥90 cm; women: ≥80 cm) and at least 2 other components: Blood pressure: ≥130/85 mm Hg TG ≥ 150 mg/dL HDL-C (men: <40 mg/dL; women: <50 mg/dL) FBG ≥ 100 mg/dL	Serum phosphorus level (3.80 ± 0.63 mg/dL (age < 40 years); 4.01 ± 2.34 mg/dL (40–60 years); 4.17 ± 1.389 mg/dL (≥60 years))	Serum phosphorus level was associated with incidence of MetS (hazard ratio (HR) = 1.39; 95% CI 1.11–1.74) and diabetes mellitus (HR = 1.49, 95% CI 1.15–1.92) in elderly aged >60 years. Serum phosphorus level was associated with increased waist circumference (HR = 1.18; 95% CI 1.06–1.31), HDL-C (HR = 1.27; 95% CI 1.07–1.50), TG (HR = 1.41; 95% CI 1.15–1.72), and FBG (HR = 1.32; 95% CI 1.14–1.53).	[28]
Terzi et al. (2015)	Prospective case–control study	Postmenopausal women with or without MetS (*n* = 230, age: 45–65 years)	Fulfilment of central obesity (waist circumference, men: ≥90 cm; women: ≥80 cm) and at least 2 other components: Blood pressure: ≥135/85 mm Hg TG ≥ 150 mg/dL HDL-C (men: <40 mg/dL; women: <50 mg/dL) FBG ≥ 100 mg/dL	Serum phosphorus level (3.7 ± 0.6 mg/dL (non-MetS); 3.6 ± 0.4 mg/dL (MetS))	No significant difference in the serum phosphorus level between patients with and without MetS.	[15]

Abbreviation: CI, confidence interval; FBG, fasting blood glucose; HDL-C, high-density lipoprotein cholesterol; HOMA-IR, homeostatic model assessment for insulin resistance; H is no * in the table, hazard ratio; MetS, metabolic syndrome; OR, odd ratio; TC, total cholesterol; TG, triglycerides.

**Table 2:** The relationship between phosphate metabolism and obesity.

Researcher (Year)	Study Type	Subjects	Phosphorus/Phosphate-Related Parameters	Phosphorus/Phosphate-Related Outcomes	Reference
Zhukouskaya et al. (2020)	Retrospective longitudinal observational study	Children with and without X-linked hypophosphatemia (*n* = 172, age: 5–20 years)		Children with X-linked hypophosphatemia had increased prevalence of overweight or obesity than the general population.	[29]
Celik & Andiran (2011)	Case control study	Normal and obese children (*n* = 177, age: 6–12 years) and adolescents (*n* = 121, age: 12–16 years)	Serum phosphate level (4.8 ± 0.4 mg/dL (obese children); 5.1 ± 0.5 mg/dL (control–children); 4.4 ± 0.5 mg/dL (obese adolescents); 4.5 ± 0.6 mg/dL (control–adolescents))	Serum phosphate level was lower in the obese children than controls. Serum phosphate level did not differ in obese adolescents and controls.	[30]
Håglin et al. (2001)	Cross-sectional study	Men (*n* = 993, age: 50.8 ± 9.4 years) and women (*n* = 1272, age: 50.1 ± 10.7 years)	Serum phosphate level (0.98 ± 0.21 mmol/L (men); 1.06 ± 0.22 mmol/L (women))	Serum phosphate level was inversely correlated with body weight and BMI in women.	[32]
Håglin et al. (2014)	Cross-sectional study	Diabetic and non-diabetic men and women (*n* = 2504, age: 50.4 ± 10.1 years)	Serum phosphate level (0.98 ± 0.20 mmol/L (men); 1.05 ± 0.21 mmol/L (women))	Serum phosphate level was inversely correlated with BMI in women.	[31]
Ayoub et al. (2015)	Double-blind, randomised, placebo-controlled trial	Adults with BMI of ≥25 kg m^−2^ and normal kidney function (*n* = 63, age: 18–45 years)	Phosphorus supplementation (375 mg/day) for 12 weeks	Body weight, BMI, waist circumference, and subjective appetite scores were lower in the phosphorus-supplemented group than placebo.	[33]
Assaad et al. (2019)	Randomised blinded cross-over study	Lean (*n* = 8) and obese (*n* = 7) male subjects (age: 20–29 years)	Phosphorus supplementation (500 mg/day)	Phosphorus supplementation with meal increased postprandial energy expenditure of both lean and obese subjects.	[34]

Abbreviation: BMI, body mass index.

**Table 5:** The relationship between phosphate metabolism and dyslipidaemia.

Researcher (Year)	Study Type	Model/Subjects	Phosphorus/Phosphate-Related Parameters	Phosphorus/Phosphate-Related Outcomes	Reference
Tanaka et al. (2013b)	Animal experimentation	C57BL/6J mice	Phosphate-restricted (0.1%) or phosphate-sufficient diet (1.2%) with or without 2% cholesterol	Phosphate restriction increased liver weight and hepatic lipid accumulation. Plasma phosphate level was inversely correlated with TC. Phosphate restriction decreased CYP7A1, HMGC-R, LDL-R, SCD1, LXRα, PPAR-α, and PPAR-γ.	[64]
Tanaka et al. (2013a)	Animal experimentation	Npt2a^−/−^ or wild type mice	Diet with or without 2% cholesterol	Npt2a^-/-^ mice had higher TC, LDL-C and HDL-C than wild type mice. High cholesterol diet increased TC, LDL-C and HDL-C in wild type mice, but not in Npt2a^−/−^ mice	[65]
Grundmann et al. (2020)	Animal experimentation	Male Ldlr^−/−^ mice	Diet containing adequate (0.3%) or high (1.5%) phosphorus with adequate (1000 IU/kg) or low (50 IU/kg) vitamin D	Mice fed with high phosphorus diet had lower TG, non-esterified cholesterol, cholesteryl esters than those fed with adequate phosphorus diet.	[66]
Abuduli et al. (2016)	Animal experimentation	Male Sprague-Dawley rats	Diet containing low (0.2%), normal (0.6%), or high (1.2%) phosphate	Rats fed with high phosphate diet had lower visceral fat accumulation and non-esterified fatty acid. High phosphate diet suppressed SREBP-1c, FAS, and ACC but did not cause any change in hepatic fat oxidation. High phosphate diet increased UCP1 and PGC-1α in brown adipose tissue.	[7]
Ditscheid et al. (2005)	Placebo-controlled, double-blind, cross-over study	Young healthy volunteers (*n* = 31, age: 21–29 years)	Bread incorporated with pentacalcium hydroxy-triphosphate (1060 mg calcium; 490 mg phosphorus), 4 weeks	Supplementation of bread containing pentacalcium hydroxy-triphosphate decreased TC, LDL-C, and LDL-C:HDL-C ratio. Supplementation of bread containing pentacalcium hydroxy-triphosphate increased excretion of cholesterol and bile acid, but did not alter the excretion of total neutral sterols.	[67]
Hazim et al. (2014)	Pilot cross-over study	Healthy male subjects (*n* = 8, age: 19.25 ± 0.41 years)	Phosphorus supplementation (500 mg)	Phosphorus supplementation did not cause any change in non-esterified fatty acid and TG. Phosphorus supplementation increased postprandial ApoB48 but decreased ApoB100.	[39]
Håglin et al. (2014)	Cross-sectional study	Diabetic and non-diabetic men and women (*n* = 2504, age: 50.4 ± 10.1 years)	Serum phosphate level (0.98 ± 0.20 (men); 1.05 ± 0.21 (women))	Serum phosphate level was directly correlated with cholesterol in women.	[31]

Abbreviation: ACC, acetyl-CoA carboxylase; ApoB48, apolipoprotein B48; ApoB100, apolipoprotein B100; CYP7A1, cholesterol 7 alpha-hydroxylase; FAS, fatty acid synthase; HDL-C, high density lipoprotein cholesterol; HMGC-R, 3-hydroxyl-3-methylglutaryl-coenzyme A reductase; LDL-C, low density lipoprotein cholesterol; Ldlr^-/-^, low-density lipoprotein receptor knockout; LDL-R, LDL-receptor; LXRα, liver X receptor alpha; Npt2a^-/-^, sodium-dependent phosphate co-transporter knockout; PGC-1α, peroxisome proliferator-activated receptor-γ coactivator-1α; PPAR-α, peroxisome proliferator-activated receptor-alpha; PPAR-γ, peroxisome proliferator-activated receptor-gamma; SCD1, stearoyl-CoA desaturase-1; SREBP-1c, sterol regulatory element-binding protein-1c; TC, total cholesterol; TG, triglycerides; UCP1, uncoupling protein 1.

## Data Availability

Not applicable.
